# Arsenomics: omics of arsenic metabolism in plants

**DOI:** 10.3389/fphys.2012.00275

**Published:** 2012-07-23

**Authors:** Rudra Deo Tripathi, Preeti Tripathi, Sanjay Dwivedi, Sonali Dubey, Sandipan Chatterjee, Debasis Chakrabarty, Prabodh K. Trivedi

**Affiliations:** Council of Scientific and Industrial Research-National Botanical Research Institute (CSIR-NBRI)Lucknow, India

**Keywords:** arsenic, arsenomics, transcriptomics, proteomics, metabolomics

## Abstract

Arsenic (As) contamination of drinking water and groundwater used for irrigation can lead to contamination of the food chain and poses serious health risk to people worldwide. To reduce As intake through the consumption of contaminated food, identification of the mechanisms for As accumulation and detoxification in plant is a prerequisite to develop efficient phytoremediation methods and safer crops with reduced As levels. Transcriptome, proteome, and metabolome analysis of any organism reflects the total biological activities at any given time which are responsible for the adaptation of the organism to the surrounding environmental conditions. As these approaches are very important in analyzing plant As transport and accumulation, we termed “Arsenomics” as approach which deals transcriptome, proteome, and metabolome alterations during As exposure. Although, various studies have been performed to understand modulation in transcriptome in response to As, many important questions need to be addressed regarding the translated proteins of plants at proteomic and metabolomic level, resulting in various ecophysiological responses. In this review, the comprehensive knowledge generated in this area has been compiled and analyzed. There is a need to strengthen Arsenomics which will lead to build up tools to develop As-free plants for safe consumption.

## Introduction

Arsenic (As) is ubiquitously present in the environment and highly toxic to all forms of life. Currently, the environmental fate and behavior of As is receiving increased attention due to the As crisis in South-East Asia. In recent decades, millions of people have suffered from As poisoning as a result of drinking As-contaminated water extracted from shallow tube wells (Christen, 2001). Large areas of Bangladesh, West Bengal and other states in India and Vietnam rely on As-contaminated ground-water for irrigation of staple crops such as rice (Nickson et al., 1998; Berg et al., 2001; Christen, 2001; Abedin et al., 2002). Consequently, in addition to exposure through drinking-water, people are being exposed to As through ingestion of food, which has been contaminated by irrigation with As rich water. A third potential route of exposure is from livestock and their products, where livestock have been fed on As-contaminated vegetation. Understanding how As is taken up by plants and subsequently accumulated in different plant parts is essential for estimating the risks posed by As-contaminated soils to humans and wildlife populations. As is released into the environment in both inorganic and organic forms. Arsenate [As(V)] and arsenite [As(III)] are the inorganic and more predominant phytoavailable forms of As in soil solution, as well as most common As species in crop plants. Organic As species such as monomethylarsenic acid and dimethylarsonic acid as also present in the environment to a lesser degree (Tripathi et al., 2007; Zhao et al., 2010). Arsenate and phosphate are chemically similar and arsenate acts as a phosphate analog, thereby being transport into the cell via the phosphate transporters (Meharg and Macnair, 1991). Through a series of As(V) and Pi transport studies, it was concluded that suppression of the high affinity Pi uptake system decreases the uptake of As(V) (Meharg and Macnair, 1992; Clark et al., 2000; Meharg and Hartley-Whitaker, 2002; Lee et al., 2003). In contrast, As(III) and undissociated methylated As species are transported through the nodulin 26-like intrinsic (NIP) aquaporin channels (Zhao et al., 2010; Mosa et al., 2012). Both inorganic forms of As are highly toxic as As(V) interferes with phosphate metabolism (such as phosphorylation and ATP synthesis) and As(III) binds to vicinal sulfydryl groups of proteins affecting their structures or catalytic functions (Tripathi et al., 2007; Zhao et al., 2010). Exposure to As(V) generates reactive oxygen species (ROS) in plant tissues, which induces oxidative stresses such as lipid peroxidation (Ahsan et al., 2008; Tripathi et al., 2012). Exposure to As(III) also enhances activity of a number of enzymes involved in the antioxidant responses (Requejo and Tena, 2005; Rai et al., 2011). A number of genes or enzymes involved in glutathione synthesis and metabolism for As sequestration are upregulated in rice seedlings exposed to As(V) (Ahsan et al., 2008; Norton et al., 2008a). This probably reflects a higher demand for GSH under As stress. Arsenate is readily reduced to As(III) in planta, which is detoxified by complexation with either thiol-rich peptides such as reduced glutathione (GSH) and phytochelatins (PCs) or vacuolar sequestration (Zhao et al., 2010) or a combination of both. To reduce the As uptake through consumption of contaminated plant foods, understanding the mechanisms of As uptake and detoxification is a prerequisite. PCs are GSH derived peptides that chelated As and participate in first step of As detoxification. Recent findings demonstrated that AtABCC1 and AtABCC2 are major vacuolar PC-As transporters (Song et al., 2010) in *Arabidopsis thaliana*. In the hyperaccumulator plant *Pteris vittata*, As(V) once taken up by the root is reduced to As(III) which is then transported to the lamina of the frond where it is stored as free As(III) (Zhao et al., 2008). Recently the vacuolar transporter (ACR3) essential for As(III) transport and As tolerance has been identified in the vacuolar membrane of the gametophyte of *P. vittata* (Indriolo et al., 2010). The yeast vacuolar transporter Ycf1p, a member of the ATP-binding cassette (ABC) superfamily, confers As(III) resistance by transporting glutathione-S-conjugated arsenite [As(III)-(GS)_3_] into the vacuole (Ghosh et al., 1999). The small duck weed *Wolfia globosa* accumulates 10 times more As than *Azolla* species, this higher accumulation is attributed to the lack of a root to frond translocation barrier (Zhang et al., 2009). In a survey of aquatic and terrestrial vegetation of West Bengal (India), high root to shoot As translocation (i.e., translocation factor (TF) > 1.0) was observed for *Phyllanthus amarus* (Tripathi et al., 2011). A detailed account of *P. vittata* and other ferns as hyperaccumulators involving higher translocation has been reviewed (Zhao et al., 2010). Among the crop plants, rice is much more highly efficient in As accumulation than other cereals, such as wheat and barley (Williams et al., 2007; Su et al., 2010). The higher accumulation is due to enhanced As bioavailability (due to being grown in anaerobic paddy conditions), and transport of As(III) through highly efficient Si transport in rice (Ma et al., 2008; Zhao et al., 2010). Enhanced accumulation of As in rice is a potentially an important route of human exposure to As, especially in populations with a rice-based diet. However, As toxicity varies greatly with species. For example, As concentrations in fish and shellfish can reach 10 mg kg^−1^ (Schoof et al., 1999) and are approximately 10–100 times the levels found in rice grain. However, most As in seafood is present in organic form such as arsenobetaine and arsenocholine that are considered to be nontoxic to humans [acute toxicity~103 times less than inorganic As (As_*i*_)]. Four species of As are commonly reported in rice grain; As(III), As(V), monomethylarsonic acid (MMA), and dimethylarsenic acid (DMA). The dominant species are usually As(III) and DMA, although the analysis for As(III) and As(V) is often jointly reported as As_*i*_' (Williams et al., 2005). The As_*i*_ content in rice grains can vary from 10–90% of total As (Heikens et al., 2007), but the reason for this variability has not been established. Discussion of the health risk of As in rice has largely been based on its As_*i*_ content because these species have generally been considered to be more toxic than MMA and DMA (NRC, 1999) and can be directly compared to As in drinking water, assuming equal bioavailability of As_*i*_ in the rice matrix and in water. The percentage of As_*i*_ in rice grain has been shown to vary by global distribution. Rice produced in the U.S. contained a mean of 42% As_*i*_ compared to 60 and 80% for European and Bangladesh/Indian rice, respectively, (Williams et al., 2005). Another study (Zavala et al., 2008) made the suggestion that methylation of As occurs within rice and that genetic differences lead to the characterization of two type rice such as DMA and Inorganic As types. Rice from the U.S. was predominantly the DMA type, whereas rice from Asia and Europe was the Inorganic As type, suggesting that the consumption of DMA rice type has lesser human health risk than the inorganic As rice type. However, other differences in the soil environment may be important. Latest work that demonstrates that plants are unable to methylate As_*i*_ (Lomax et al., 2012) and instead take up methylated As produced by microorganisms in the soil environment. Therefore it is unlikely for genetic differences to exist for the methylation of As_*i*_, however a number of studies have demonstrated that there is genetic differences in the accumulation of total As in rice grains (Williams et al., 2005, 2007; Dwivedi et al., 2010, 2012).

Though considerable progress has been made in elucidating the mechanism of plant As uptake and metabolism including tolerance and toxicity aspects, there is a pressing need to summarize these outcomes in terms of omic technologies. A better understand of the mechanisms involved in assimilation and metabolization of As will led to development of mitigation strategies against this widespread contamination of the food chain. Here, we focused on the harmonized response of the plant in terms of omic technologies, which is the nontargeted identification of various products (transcripts, proteins, and metabolites) during As stress in a specific biological sample. The aim of this review is to (a) provide an in-depth overview about genomic, transcriptomic, proteomic, and metabolomic approaches of plant for both As tolerance and detoxification, (b) explore how genomic, transcriptomic, proteomic, and metabolomic networks can be connected to underlying plant mechanisms during As stress, and (c) discuss the need to investigate integrative biochemical networks in As metabolism. Here the As-mediated alteration in plant biological process is summarized in terms of genomics, proteomics, and metabolomics in the following sections.

## Genomic and transcriptional regulation during arsenic stress

### Arsenic transporters

Analysis of the rice and *Arabidopsis* genomes revealed presence of at least 13 and 9 members of the phosphate transporter (*Pht1*) family in these species, respectively, (Okumura et al., 1998; Mudge et al., 2002; Paszkowski et al., 2002). Shin et al. (2004) indicated that *Pht1;1* and *Pht1;4* mediate a significant proportion of the As(V) uptake in *Arabidopsis*. Arsenite is the dominant As species in reducing environments, such as flooded paddy soils (Meharg and Jardine, 2003). Bienert et al. (2008) showed that the Nodulin26-like Intrinsic Proteins (NIPs) *OsNIP2;1* and *OsNIP3;2* from rice are bi-directional As(III) channels. Direct transport assays using yeast cells confirmed their ability to facilitate As(III) transport across cell membranes. Ma et al. (2008) reported that two different types of transporters mediate transport of As(III), from the external medium to the xylem. Transporters belonging to the NIP subfamily of aquaporins in rice are permeable to As(III) but not to As(V). Mutation in *OsNIP2;1* (*Lsi1*, a silicon influx transporter) significantly decreased As(III) uptake. Furthermore, in the rice mutants defective in the silicon efflux transporter, *Lsi2*, As(III) transport to the xylem and accumulation in shoots and grain decreased substantially. Mutation in *Lsi2* had a much greater impact on As accumulation in shoots and grains in field-grown rice than *Lsi1*. The efficient Si uptake pathway in rice also allows inadvertent passage of As(III), thus explaining why rice is efficient in accumulation of As (Ma et al., 2008). However, knockout of these transporters may not provide solution to As accumulation, as NIPs facilitate the uptake of vital nutrients such as boron and silicon (Ma et al., 2008).

### Gene mapping for arsenic and other elements accumulation

An As tolerance gene has been identified and mapped to chromosome 6 in rice. The collocation of As tolerance gene with a phosphate uptake QTL in another population of rice provided circumstantial evidence for a mechanism involving the behavior of As(V) as a phosphate analog, in agreement with the behavior of an As resistance gene found in populations of *Holocus lanatus* (Dasgupta et al., 2004). Recently, the genetic mapping of the tolerance of root growth to As(V) using the Bala X Azucena population suggested involvement of epistatic interaction involving three major genes, two on chromosome 6 and one on chromosome 10. The study provided physiological evidence that genes related to phosphate transport is unlikely to be behind the genetic loci conferring tolerance (Norton et al., 2008b). However, Zhang et al. (2007) measured As accumulation in roots and shoots at the seedling stage and brown rice at maturity of the parental cultivars (CJ06/TN1) and their double haploid lines after exposure to 210 mg As/kg As in a pot experiment. Four QTLs for As concentrations were detected in the genetic map. At the seedling stage, one QTL was mapped on chromosome 2 for As accumulation in shoots which explained 24% of the observed phenotypic variance and another QTL for As accumulation in roots was detected on chromosome 3. At maturity, two QTLs for As accumulation in grains were found on chromosomes 6 and 8, which explained 26 and 35% of the phenotypic variance, respectively. This study also showed that the QTL on chromosome 8 was identified for As concentrations in grain at maturity and shoot phosphorus (P) concentrations at seedling stage. A study targeting genetic mapping of the rice ionome in leaves and grains identified QTLs for 17 elements including As, Se, Fe, and Cd. In general, there were no QTL clusters suggesting independent regulation of each element. An epistatic interaction for grain As appears promising to decrease the concentration of this carcinogenic metalloid in the rice grain (Norton et al., 2010). Further, these workers while identifying QTL for rice grain element composition on an As impacted soil from China, found a correlation between flowering time and number of element concentrations in grains, which revealed co-localization between flowering time QTLs and grain element QTLs. Therefore, this study concluded that flowering time is one of the major factor that controls As and other element accumulation in the rice grain. Besides, an epistatic interaction for grain As also looks promising to decrease the concentration of this carcinogen (Norton et al., 2012). Recently, As concentrations in different tissues of maize was analyzed using a RIL population (Ding et al., 2011). In this study, three QTLs for As concentration in leaves were mapped on chromosomes 1, 5, and 8, respectively. For As concentration in the bracts, two QTLs were identified, which accounted for around 10% of the observed phenotypic variance. For As concentration in the stems, three QTLs were detected with around 8–15% phenotypic variance. Three QTLs were identified for kernels on chromosomes 3, 5, and 7, respectively, with around 9–11% phenotypic variance. Only one common chromosomal region between SSR marker bnlg1811 and umc1243 was detected for QTLs *qLAV1* and *qSAC1*. These results demonstrated that the As accumulation in different tissues in maize was controlled by different molecular mechanisms.

### Differential expression of various genes during arsenic stress

The effect of As exposure on genome-wide expression was also examined in rice by different groups (Norton et al., 2008a; Chakrabarty et al., 2009; Yu et al., 2012). A group of defense and stress-responsive transporters including; sulphate transporters, heat-shock proteins, metallothioneins, multidrug and toxic compound extrusion (MATE) transporters, glutathione-S-transferase, multidrug resistance proteins, glutathione conjugated transportes, metal transporter viz. NRAMP1, and genes of sulfate-metabolizing proteins were commonly upregulated in rice during As(V) stress in both the studies (Norton et al., 2008a; Chakrabarty et al., 2009). In contrast, phosphate transporter, zinc transporter, aquaporin gene, amino acid transporters, and peroxidases (PODs) were commonly downregulated, however, some gene like cytochrome P450, oxidoreductase were differentially regulated in rice seedling challenged with As(V). Yu et al. (2012) first used high-throughput sequencing technology to study transcriptomes of plant response to As stress. Overall, by genome-wide transcriptome and miRNA analyses in rice seedlings treated with As(III), they found a large number of potentially interesting genes in relation to As(III) stress, especially As(III)-responsive transporters and TFs (transcription factors). The change in the expression of genes related to lipid metabolism and phytohormone pathways after As(III) exposure was striking, indicating that rice invests more energy and resources into immediate defense needs than into normal growth requirements.

Arsenate and As(III) stresses modulated metabolic pathways networks affecting various physiological processes necessary for plant growth and development (Chakrabarty et al., 2009). Arsenate stress led to upregulation or downregulation of additional genes in comparison to As(III), but one glutaredoxin (Os01g26912) is expressed specifically in the AsIII-treated shoots. In one of our studies, four rice genotypes responded differentially under As(V) and As(III) stress in terms of gene expression and antioxidant defenses (Rai et al., 2011). Arsenate challenge leads to altered gene expression in a large number of genes involved in classical oxidative stress response, however, phytochelatin synthase (PCS) and arsenic reductase (AR) levels were not altered significantly. This study suggested that the rice cultivar, IET-4786, is very sensitive to As stress due to reduction of both sulphate assimilation pathway and antioxidant defense enzymes in As detoxification in contrast to the tolerant cultivar, Triguna. The other two varieties (PNR-546 and IR-36) exhibited intermediary responses involving regulation of these genes during As stress (Rai et al., 2011). The differential expression pattern of sulphate transporters were observed for the varieties after As(V) exposure (Kumar et al., 2011). Most of the sulphate transporters studied were down-regulated in the sensitive variety IET-4786 after exposure to As(V). Interestingly, differential alternative splicing for *OsSultr1;1* was also observed for all the lines after As exposure. A decrease in accumulation was observed for the largest splice variant containing both the introns at higher concentrations of As(V) and As(III). In the case of sensitive rice variety, accumulation of the entire splice variant decreased with increasing concentration of As(V). These results clearly suggest that the expression of sulphate transporter gene family with respect to heavy metal stress is regulated differentially in different cultivars.

However, several common processes were affected by As(V) and As(III). Differential expression of several genes that showed the highest contrast in a microarray analysis was validated by following the quantitative changes in the levels of individual transcripts following challenge with As(V)and As(III). Abercrombie et al. (2008) first studied the transcriptional response of a dicot plants, *Arabidopsis thaliana*, to As(V) stress using oligonucleotide microarrays. The study suggested that As(V) stress strongly induces Cu/Zn superoxide dismutase activity (SOD), but represses the production of Fe SOD. The study also suggested involvement of several other genes related to antioxidant systems, various transcription factors, tonoplast proteins, and proteins associated with cell wall growth. A comparative biochemical and transcriptional profiling of two contrasting varieties (tolerant and sensitive to As) of *Brassica juncea* indicated upregulation of sulfate transporters and auxin and jasmonate biosynthesis pathway genes, whereas, there was downregulation of ethylene biosynthesis and cytokinin responsive genes in As tolerant plant in contrast to sensitive plant (Srivastava et al., 2009). In another experiment, Paulose et al. (2010) studied expression profiling of dicot *Crambe abyssinica* under As(V) stress using PCR-Select Suppression Subtraction Hybridization (SSH) approach. Their study revealed novel insights into the plant defense mechanisms, and the regulation of genes and gene networks in response to arsenate toxicity. The differential expression of transcripts encoding glutathione-S-transferases (GSTs), antioxidant As_*i*_ genes, sulfur metabolism genes, heat-shock proteins, metal transporters, and enzymes in the ubiquitination pathway of protein degradation, as well as several unknown novel proteins serve as molecular evidence for the physiological responses to As(V) stress in plants.

A comparative evaluation of As responsive genes in monocots and dicots offered interesting observations (Table 1). Various PODs genes always showed a down regulation (2–24 fold) in monocots, however, upregulation of two PODs was recorded in dicots. MT like proteins was upregulated in monocots. Five ferrodoxins were down regulated in rice but not in *A. thaliana*. Peptidyl prolyl cis-trans isomerase was highly upregulated in rice in contrast to *A. thaliana*. Interestingly, 9 GSTs were upregulated in rice, whereas only one was observed to be upregulated in *Arabidopsis*. At the same time, in both the plants 2 GSTs were downregulated. While most of the cytochrome P450 were upregulated in rice, none of these were upregulated in *Arabidopsis*. In contrast to *A. thaliana*, the germin like protein was upregulated in rice. Many genes encoding ferritins, ZFP (Zinc finger protein), patatin, acid phosphates, serine/threonine proteine kinases, integral membrane proteins, and hydrolases always were down regulated in both monocot and dicots (Abercrombie et al., 2008; Chakrabarty et al., 2009). The data suggests that though there are common mechanisms for As metabolisms in dicot and monocot plants, differential mechanisms may also exists.

**Table 1:** **Sets of differentially regulated genes in monocot (*O. sativa*) and dicot (*A. thaliana*) plants during “As” stress**.

**Description of gene**	**Monocot**		**Dicot**	
	**Locus**	**Fold change (+/−)**	**Locus**	**Fold change (+/−)**
Peroxidase	Os05g04410	(−) 4.14	At5g64100	(+) 2.50
	Os05g04450	(−) 5.94	At1g05250	(+) 1.90
	Os03g36560	(−) 3.69	At5g17820	(+) 1.68
	Os07g01410	(−) 5.74	At1g05240	(+) 2.05
	Os04g59160	(−) 12.60	At3g49120	(−) 1.77
	Os04g59160	(−) 8.33	At5g64120	(−) 1.84
	Os04g59260	(−) 3.67	At4g25980	(−) 1.52
	Os03g25330	(−) 3.73		
	Os03g25340	(−) 4.63		
	Os03g55410	(−) 2.33		
	Os05g06970	(−) 2.98		
	Os07g31610	(−) 10.54		
	Os04g59190	(−) 34.18		
	Os05g06970	(−) 2.98		
	Os07g31610	(−) 10.54		
	Os04g59190	(−) 34.18		
	Os10g39160	(−) 7.71		
	Os01g18970	(−) 10.62		
	Os07g44480	(−) 24.42		
	Os07g44460	(−) 7.04		
	Os07g44460	(−) 12.11		
Metallothionein-like protein 1	Os12g38064	(+) 2.60	At1g07600	(+) 1.67
	Os12g38300	(+) 2.44		
	Os04g44250	(+) 3.42		
Ferredoxin, chloroplast	Os08g01380	(−) 3.49	At1g10960	(+) 1.53
	Os08g01380	(−) 3.63		
	Os08g01380	(−) 2.75		
	Os01g25484	(−) 3.67		
	Os01g64120	(−) 4.43		
Glycosyl hydrolase family 1 protein	Os02g20360	(+) 2.62	At3g09260	(+) 1.67
	Os01g54300	(−) 3.50		
Peptidyl prolyl cis-trans isomerase	Os04g28420	(+) 18.72	At3g62030	(+) 1.64
Hypothetical protein	Os08g45120	(+) 5.76	At2g06480	(+) 1.58
	Os03g07510	(+) 3.14		
	Os08g04560	(+) 3.74		
	Os11g16990	(−) 7.55		
Glutathione S-transferase	Os01g72140	(+) 2.53	At1g78370	(+) 1.68
	Os01g49710	(+) 3.40	At1g02930	(−) 2.10
	Os10g20350	(+) 3.31	At1g02920	(−) 2.88
	Os753122	(+) 2.68		
	Os03g13390	(+) 4.01		
	Os06g44010	(+) 2.48		
	Os07g23570	(+) 3.29		
	Os03g46110	(+) 4.91		
	Os06g13190	(+) 2.41		
	Os01g27630	(−) 6.29		
	Os01g27390	(−) 2.46		
Catalase	Os03g03910	(−) 4.52	At1g20620	(−) 1.59
Cationic peroxidase	Os01g18950	(−) 20.77	At4g25980	(−) 1.52
Lipoxygenase	Os08g39850	(+) 4.21	At1g72520	(−) 2.41
	Os03g49260	(−) 2.66		
Cytochrome P450 83B1	Os03g55240	(+) 3.07	At4g31500	(−) 1.71
	Os08g39730	(+) 6.09	At3g48520	(−) 1.56
	Os02g36190	(+) 2.95		
	Os01g43740	(+) 8.51		
	Os01g38110	(+) 7.76		
	Os01g43774	(+) 30.16		
	Os01g50170	(+) 2.80		
	Os03g57640	(+) 3.00		
	Os09g27260	(+) 8.10		
	Os03g26210	(+) 2.49		
	Os09g10340	(−) 2.74		
Germin-like protein	Os03g06970	(+) 7.19	At5g39160	(−) 1.51
			At5g39190	(−) 2.13
Ferritin	Os12g01530	(−) 3.45	At5g01600	(−) 1.78
	Os12g01530	(−) 3.29	At3g56090	(−) 1.52
	Os11g01530	(−) 4.58		
Zinc finger protein	Os06g04920	(−) 2.72	At3g46090	(−) 1.51
			At3g46080	(−) 1.59
			At5g27420	(−) 1.75
Acid phosphatase	Os07g48320	(−) 3.15	At3g17790	(−) 1.62
Gycosyl hydrolase family 17 protein	Os02g20360	(−) 2.62	At3g55430	(−) 1.53
			At4g31140	(−) 1.71
			At4g19810	(−) 1.96
			At5g20250	(−) 1.52
Xloglucan endotransglucosylase/ hydrolase	Os02g17900	(−) 17.69	At4g30280	(−) 1.63
	Os02g17880	(−) 10.85	At4g14130	(−) 2.00
	Os06g22919	(−) 10.29	At5g57560	(−) 1.68
Patatin	Os08g37250	(−) 3.47	At2g26560	(−) 1.81
	Os08g37250	(−) 4.58		
Serine/threonine protein kinase	Os02g43370	(−) 3.31	At3g08720	(−) 1.55
	Os12g05394	(−) 3.27		
	Os09g12240	(−) 3.49		
Integral membrane family protein	Os04g45520	(−) 18.34	At4g15610	(−) 1.58
NAC domain-containing protein	Os10g21630	(+) 5.81	At5g08790	(−) 1.53
	Os02g15340	(−) 3.71		

Compilation of data based on monocot O. sativa (Chakrabarty et al., 2009) and dicot (A. thaliana) (Abercrombie et al., 2008) plants during arsenate stress. Upregulated (+), Down regulated (−).

### Arsenic tolerance in transgenic plants

Gasic and Korban (2007) reported that overexpression of the *A. thaliana* phytochelatin synthase *AtPCS1* gene in Indian mustard enhanced tolerance to As and Cd. Similarly, Song et al. (2010) showed that in the absence of two ABCC-type transporters, AtABCC1 and AtABCC*2*, *A. thaliana* is extremely sensitive to As and As-based herbicides. Heterologous expression of these ABCC transporters in phytochelatin (PC)-producing *Saccharomyces cerevisiae* enhanced As tolerance and accumulation. Furthermore, membrane vesicles isolated from yeasts exhibited a pronounced As(III)–PC_2_ transport activity. Recently, Indriolo et al. (2010) characterized two genes from *P. vittata* (*ACR3* and *ACR3;1*), which encode proteins similar to the ACR3 As(III) effluxer of yeast. It was also showed that *ACR3* is localized to the vacuolar membrane in gametophytes, indicating that it likely effuxes As(III) into vacuoles for sequestration. A single copy of *ACR3* genes is present in moss, other ferns and gymnosperms but absent in flowering plants. The absence of *ACR3* genes in both monocots and eudicots may preclude their ability to hyperaccumulate As, which is consistent with the observation that no As-hyperaccumulating angiosperm has ever been identified. Why the highly conserved *ACR3* gene was lost in the angiosperm lineage may never be known, but its loss coincides with their reliance on insects and other animals for pollination and fruit dispersal. On the other hand, the selection of As hyperaccumulation in *P. vittata*, may have evolved as a deterrent and in response to herbivores, as it has recently been shown that insects avoid eating *P. vittata* grown in the presence of As, preferring those not grown in As (Rathinasabapathi et al., 2007). In another study, examining heterologous expression of the yeast As(III) efflux system, ACR3 was cloned from yeast and transformed into wild-type and nip7;1 *Arabidopsis* (Ali et al., 2012). At the cellular level, all transgenic lines showed increased tolerance to As(III) and As(V) and a greater capacity for As(V) efflux. With intact plants, three of four stably transformed lines showed improved growth, whereas only transgenic lines in the wild-type background showed increased efflux of As(III) into the external medium. The presence of ACR3 hardly affected tissue As levels, but increased As translocation to the shoot. Expressing *Saccharomyces cerevisiae ScACR3* in rice enhanced As(III) Efflux and also reduced As accumulation in rice grains (Duan et al., 2012). In the transgenic lines, As concentrations in shoots and roots were about 30% lower than in the wild type, while the As translocation factors were similar between transgenic lines and the wild type. The roots of transgenic plants exhibited significantly higher As efflux activities than those of the wild type. Importantly, ScACR3 expression significantly reduced As accumulation in rice straws and grains. When grown in flooded soil irrigated with As(III)-containing water, the As concentration in husk and brown rice of the transgenic lines was reduced by 30 and 20%, respectively, compared with the wild type. This study reports a potential strategy to reduce As accumulation in the food chain by expressing heterologous genes in crops. Recently, Sundaram et al. (2009) overexpressed the *P. vittata* glutaredoxin *PvGRX5* gene in *Arabidopsis*. It was observed that two lines of *A. thaliana* constitutively expressing *PvGrx5 cDNA* were significantly more tolerant than vector control and wild-type lines on the basis of germination, root growth and whole plant growth under As stress. A study was conducted by Grispen et al. (2009) by overexpressing *AtMT2b* in *Nicotiana tabacum*. It was observed that the highest *AtMT2b* expressing line exhibited a significant decrease in As accumulation in roots, but an increased accumulation in shoots, while the total amount of As taken up remained unchanged. This clearly suggested that *AtMT2b* expression may enhance As root to shoot transport. Rice transgenics overexpressing As(III)-S-adenosyl methyl transferase (*arsM*) has been found to methylate As, and gave 10 fold higher volatile arsenical, maintaining low As levels in rice seed along with MMA(V) and DMA(V) in the roots and shoots of transgenic rice (Meng et al., 2011). The upregulation of Met synthase and AdoMet synthetase could correlate with As(III) methylation. Although the amounts of methylated As were small, this may offer a new stratagem for As phytoremediation. Shukla et al. (2012) demonstrated that transgenic tobacco plants expressing *Ceratophyllum demersum* phytochelatin synthase *CdPCS1* had a several fold increase in PC content, precursor non protein thiols, and enhanced accumulation of As without significant decrease in plant growth.

## Proteome modulation during arsenic stress

During the last decade, several groups have made use of transcriptome analysis to investigate the expression patterns of genes in plants under As stress (Abercrombie et al., 2008; Norton et al., 2008a; Chakrabarty et al., 2009). Such analysis of gene expression at the mRNA level has enhanced our understanding of the responses of plants to As stress. However, transcriptional analysis has a number of limitations (Rose et al., 2004), including poor correlations between changes in the expression of mRNAs and those of their corresponding proteins (Gygi et al., 1999). Though correlation between mRNA and protein has been noticed (Joosen et al., 2007; Li et al., 2007), protein expression is also regulated at the translational and post-translational levels. Thus, gaining information at the translational and post-translational levels can offer deeper insights into the responses and functional interactions of proteins.

Although large numbers of physiological and biochemical analyses have been performed (Hartley-Whitaker et al., 2001; Liu et al., 2005; Williams et al., 2005; Rahman et al., 2007), little is known about As stress-elicited changes in plants at the proteome level. A summary of upregulated and downregulated proteins during As stress in different plant species is presented in Table 2. Expression patterns of maize (*Zea mays*) root and leaf proteins in response to As stress were described for the first time by Requejo and Tena (2005, 2006). They showed that 10% of detectable proteins in maize roots were differentially regulated by As; seven of the eleven proteins that were identified are involved in cellular homeostasis for redox perturbation, suggesting that oxidative stress is a major process underlying As toxicity in plants (Requejo and Tena, 2005). To gain a comprehensive understanding of the precise mechanisms underlying the toxicity of As to rice, the metabolism and defense reactions in As nontolerant plants have recently been examined by Ahsan et al. (2008). This was done by carrying out a comparative proteomic analysis of rice roots in combination with physiological and biochemical analyses. The comparative proteomic analyses identified 23 differentially expressed proteins in rice roots, including those predicted as S-adenosylmethionine synthetase (SAMS), cysteine synthase (CS), and novel proteins including tyrosine-specific protein phosphatase protein, and omega domain containing GST. These differentially expressed proteins are functionally involved in cell signaling, stress and detoxification, defense and development, and protein biosynthesis. On the basis of physiological and proteomic investigations, it is proposed that As stress in plants generates ROS, triggering signaling molecules such as jasmonic acid (JA) and S-adenosyl-L-methionine (SAM), and activating the detoxification process, which mainly involves GSH/PC biosynthesis (Ahsan et al., 2008). Interestingly, these pathways are coordinately regulated, with GSH playing a central role in the complex cellular network. Arsenite and As(V) differentially modulated physiological response in *Hydrilla verticillata*, where As(V) stress resulted in activation of the antioxidant defence system, while As(III) responses involved prevalence of As complexing PCs (Srivastava et al., 2007). Elevated levels of antioxidants and PCs were also observed in *C. demersum* during As(V) exposure, suggesting the modulation of specific proteins by As induced stress (Mishra et al., 2008). The As induced antioxidative responses were also validated by proteomic changes in germinating rice seedlings during As exposure. Further study of isozymes of SOD, APX, POD, and glutathione reductase (GR) validated that As accumulation generated oxidative stress, which was more pronounced in As(III) treatment (Shri et al., 2009). Ahsan et al. (2010) also reported the first proteome map of rice leaves under As stress along with physiological and biochemical responses. The increased activity of several proteins associated with energy metabolism, such as NADP-dependent malic enzyme, NAD-dependent formate dehydrogenase, and glyceraldehyde-3-phosphate dehydrogenase suggest that an increased amount of energy is required to adapt to As stress. However, the down-regulation of RuBisCO and chloroplast 29 kDa ribonucleoproteins might be the possible causes of the decreased photosynthesis rate under As stress. Contrary to this finding, Duquesnoy et al. (2009) identified a set of *Agrostis tenuis* leaf proteins differentially expressed in response to As exposure including a major functionally homogeneous group of enzymes including oxygen-evolving enhancer protein, RuBisCO small and large subunits, RuBisCO activase, and ATP synthase involved in the Calvin or Krebs cycle. Bona et al. (2010) also demonstrated the protein expression profile of *P. vittata* fronds in plants inoculated with one of the two AM fungi (*Gigaspora mosseae* or *Gigaspora margarita*) with and without As treatment. Up-regulation of multiple forms of glyceraldehyde-3-phosphate dehydrogenase, phosphoglycerate kinase (PGK), and enolase, primarily in *G. mosseae*-inoculated plants, suggests a central role for glycolytic enzymes in As metabolism. Moreover, a putative As transporter, *PgPOR29*, has been identified as an up-regulated protein by As treatment. Proteomics in conjunction with morphological, physiological, and biochemical variables has been employed for the first time by Pandey et al. (2011) to unravel survival strategies of the diazotrophic cyanobacterium *Anabaena* sp. PCC7120. Down-regulation of PGK, fructose bisphosphate aldolase II (FBA II), fructose 1,6 bisphosphatase (FBPase), transketolase (TK), and ATP synthase on day 1 and their significant recovery on the day 15 presumably maintained the glycolysis, pentose phosphate pathway (PPP) and turnover rate of Calvin cycle, hence survival of the test organism. Up-regulation of CAT, peroxiredoxin (Prx), thioredoxin (Trx), and oxidoreductase appears to protect the cells from oxidative stress. Appreciable induction in PC content, GST activity and transcripts of PCS, AR and As(III) efflux genes-*asr1102*, *alr1097* reiterates their role in As sequestration and shielding of the organism from As toxicity. While up-regulated metabolic and antioxidative defense proteins, PC and GST work synchronously, the *ars* genes play a central role in detoxification and survival of *Anabaena* under As stress. To elucidate the mechanisms of As resistance in the As hyperaccumulator fern *P. vittata*, a cDNA for a glutaredoxin (Grx) Pv5–6 was isolated from a frond expression cDNA library based on the ability of the cDNA to increase As resistance in *Escherichia coli*. The deduced amino acid sequence of Pv5–6 showed high homology with an *Arabidopsis* chloroplastic Grx and contained two C*XX*S putative catalytic motifs. Purified recombinant Pv5–6 exhibited glutaredoxin activity that was increased at 10 mM As(V). Site-specific mutation of Cys^67^ to Ala^67^ resulted in the loss of both GRX activity and As resistance. *PvGrx5* has an important role in regulating intracellular As(III) levels, by either directly or indirectly modulating the aquaglyceroporin (Sundaram et al., 2008).

**Table 2:** **A review of “As” modulated proteins in different plant species during proteomic analysis**.

**Plant species**	**Plant part used**	**No. of responsive proteins and proteomic analysis used**	**Research outcome**	**References**
*Z. mays*	Root	11, 2-DE, MALDI-TOF MS	Upregulation of antioxidant enzymes related proteins *viz.* SODs, GPXs, and peroxiredoxin (Prx) besides four additional, functionally heterogeneous, proteins (e.g., ATP synthase, succinyl-CoA synthetase, cytochrome P450, and guanine nucleotide-binding protein b subunit) suggest that the induction of oxidative stress is a main process underlying As toxicity in plants	Requejo and Tena, 2005
*Z. mays*	Shoot	7, 2-DE, MALDI-TOF MS	Down regulation of Translation initiation factor eIF-5A, ATP synthase, CS, malate dehydrogenase, protein kinase C inhibitor, Tn10 transposase-like protein, and guanine nucleotide binding protein during As stress	Requejo and Tena, 2006
*O. sativa*	Root	23, 2-DE, MALDI-TOF MS	GSH plays a central role in protecting cells against As stress due to synchronous function of SAMS, CS, GSTs, and GR	Ahsan et al., 2008
*O. sativa*	Leaf	6, 2-DE, MALDI-TOF MS	Down-regulation of RuBisCO and chloroplast 29 kDa ribonucleo proteins under As stress may be the possible causes of the decreased photosynthesis rate	Ahsan et al., 2010
*P. vittata* (G. mosseae-inoculated)	Frond	19, 2-DE hybrid quadrupole-TOF MS	Multiple forms of glyceraldehyde-3-phosphate dehydrogenase, phosphoglycerate kinase, and enolase, was upregulated in G. mosseae-inoculated plants, suggests a central role for glycolytic enzymes in As metabolism	Bona et al., 2010
*A. tenuis*	Leaf	2-DE hybrid quadrupole-TOF MS	prominent fragmentation of the RubisCO protein due to As toxicity	Duquesnoy et al., 2009

Other than these investigations, no proteomic analyses have been carried out on responses of dicotyledonous plants including *Arabidopsis*, to As stress. Therefore, there is a need for an extended proteomic analysis of the responses of the root systems of dicotyledonous model plants systems to As stress to gain a better understanding of the molecular basis of this response. This will allow the determination of whether monocot and dicot plants both employ the same defense mechanisms under As stress, and to identify novel As-responsive proteins for future studies.

## Metabolome modulation during arsenic stress

Despite the fact that transcriptomic approaches provide almost complete coverage and proteomics approaches are now capable of detecting most of the cellular protein complement, metabolomics is currently capable of determining only a small fraction of the metabolites found in any one cell. As well as to validate the outcome of differential transcriptomic studies along with proteomic analyses in As stressed plant, metabolome analysis is needed to investigate the unexplored properties of biological systems. The more challenging aspect of metabolomic technologies is the refined analysis of quantitative dynamics in biological systems. For metabolomics, gas and liquid chromatography coupled to mass spectrometry are well suited for coping with high sample numbers in reliable measurement times with respect to both technical accuracy and the identification and quantitation of small-molecular-weight metabolites. However to best of our knowledge, there is very limited studies have been performed to recognize the modulation of differential metabolomic pathway during As stress.

### Role of glutathione and phytochelatin during arsenic stress

In most of the studies, metabolites involved in antioxidant systems, PCs and related molecules involved in biosynthesis of PCs have been analyzed during As stress. A list of several As responsive metabolites has been mentioned in the Table 3. The raised level of some metal detoxifying thiolic ligand such as glutathione were also noticed in ferns such as *P. vittata*, *P. ensiformis* (Singh et al., 2006), aquatic plants such as *H. verticilata* and *C. demersum* (Srivastava et al., 2007; Mishra et al., 2008), and crop plants like *B. juncea* and *O. sativa* (Srivastava et al., 2009; Tripathi et al., 2012). While concomitant reduction in glutathione and S-nitrosoglutathione (GSNO) content were observed in *A. thaliana* suggesting the altered GR and S-nitrosoglutathione reductase activities during higher As exposure (Leterrier et al., 2012). Similarly induced level of PCs were also observed in *H. verticillata*, *C. demersum*, and *O. sativa* (Srivastava et al., 2007; Mishra et al., 2008; Tripathi et al., 2012) under As stress. Earlier, As-PC complexes were studied in *H. lanatus* and *P. cretica* using parallel metal(loid)-specific-ICPMS and organic-specific-ESI-MS detection systems (Raab et al., 2004). In an *in vitro* experiment using a mixture of GSH, PC_2_, and PC_3_, As preferred the formation of the As(III)-PC_3_ complex over GSH-As(III)-PC_2_, As(III)-(GSH)_3_, As(III)-PC_2_, or As(III)-(PC_2_)_2_. In *H. lanatus*, the As(III)-PC_3_ complex was the dominant complex, although GSH, PC_2_, and PC_3_ were found in the tissue extract. *P. cretica* only synthesizes PC_2_ and forms dominantly the GSH-As(III)-PC_2_ complex. In both plant species, As is dominantly in non-bound inorganic forms, with 13% being present in PC complexes for *H. lanatus* and 1% in *P. cretica*. Phytochelatin synthesis was induced upon exposure to As(V) in *P. vittata*, with only PC_2_ detected in the plant (Zhao et al., 2003). The As concentration correlated significantly with PC_2_ concentration in roots and shoots of *P. vittata*, but not with GSH. Chelation of only a small proportion (1–3%) of the As with PCs in *P. vittata* suggests that PCs play a limited role for the hypertolerance of As in *P. vittata*. Similarly, Raab et al. (2007) demonstrated the As concentration-dependent formation of As–PC complex, redistribution and metabolism of As after arrested As uptake in *Helianthus annuus*. The amount and number of As–PC complexes increased exponentially with concentration up to 13.7 μM As and As(III)–PC_3_ and GS–As(III)–PC_2_ complexes were the dominant species. In another study, Liu et al. (2010) quantified As(III)-thiol complexes and free thiol compounds in *A. thaliana* exposed to As(V). In wild-type roots, 69% of As(III) was complexed as As(III)-PC_4_, As(III)-PC_3_, and As(III)-(PC_2_)_2_ while in roots of the GSH-deficient mutant (cad2-1) and the PC-deficient mutant (cad1-3) very little of As was complexed with As(III)-PCs and As(III)-(GS)_3_, respectively. This conferred approximately 20 times more tolerance for the wild type than the mutants. These mutants showed significantly higher accumulation of As(III) in shoots and effluxed larger amount of As(III) than the wild type, suggesting that enhancing PC synthesis in roots may be an effective strategy to reduce As translocation to the edible organs of food crops. Furthermore, transgenic *Arabidopsis* expressing very high levels of the bacterial γ-glutamylcysteine synthetase (ECS) gene and PCS had several fold higher concentrations of γ-glutamylcysteine (EC), GSH, and PCs than the wild type, and show tolerance to As (Dhankher et al., 2002; Li et al., 2004).

**Table 3:** **Metabolites in different plant species during arsenic stress**.

**Metabolites studied**	**Plant species**	**Research outcome**	**References**
Valine, metheionine, leucine, alanine histidine, alanine, proline, glutamic acid, cysteine	*O. sativa*	Most of the NEEAs were increased in most of the cultivars during As stress, while EAAs were decreased in most of the cultivar	Dwivedi et al., 2010, 2012
Proline, glutamic acid, aspartic acid alanine	*S. oleracea*	Increased during As(V) stress	Pavlík et al., 2010
Proline	*O. sativa*	Induced during As(III) stress	Mishra and Dubey (2006)
Cysteine	*H. verticillata, C. demersum, B. juncea, O. sativa*	Increased during As(V) stress	Srivastava et al., 2007, 2009; Mishra et al., 2008; Tripathi et al., 2012
γ-glutamyl cysteine	*A. thaliana*	Increased synthesis during As(V) stress	Dhankher et al., 2002; Li et al., 2004
Glutathione	*A. thaliana, P. vittata, P. ensiformis, H. verticillata, C. demersum, B. juncea, O. sativa, A. thaliana*	Increased during As(V) stress except in *A. thaliana*	Dhankher et al., 2002; Li et al., 2004; Singh et al., 2006; Srivastava et al., 2007, 2009; Mishra et al., 2008; Tripathi et al., 2012
S-nitrosoglutathione	*A. thaliana*	Decreased during As(V) stress	Leterrier et al., 2012
Phytochelatins	*A. thaliana, H. verticillata, C. demersum, O. sativa*	Various species of phytochelatins were increased during As stress	Dhankher et al., 2002; Li et al., 2004; Srivastava et al., 2007; Mishra et al., 2008; Tripathi et al., 2012
Ascorbic acid	*P. vittata, P. ensiformis, O. sativa*	Increased during As(V) stress	Singh et al., 2006; Tripathi et al., 2012
Malondialdehyde	*P. vittata, P. ensiformis, H. verticillata, C. demersum, O. sativa*	Increased during As(V) stress	Singh et al., 2006; Srivastava et al., 2007; Mishra et al., 2008; Tripathi et al., 2012
Nitric oxide	*H. verticillata, O. sativa, A. thaliana*	Increased during As(V) stress	Srivastava et al., 2011; Leterrier et al., 2012; Tripathi et al., 2012
Polyamines (spermidine, spermine) and diamine (putrescine)	*T. pratense*	Polyamines increased only at lower doses but diamine increased at higher doses during As(V) stress	Mascher et al., 2002
ATP, ADP, NADH, NAD, NADPH, NADP	*H. verticillata*	Level of ATP, NADP, NADH decreased, while level of ADP, NADPH and NAD increased during As(V) exposure	Srivastava et al., 2011

### Changes in amino acid profiling

Variation in amino acid content was observed in different plant species during As exposure. Dwivedi et al. (2010) performed a simulated pot experiment, using environmentally relevant concentrations of As, analyzed the amino acid profile in grain of various rice genotypes. This study demonstrated that Specific As Uptake (SAU, μg g^−1^dw), which indicates the ability of As uptake by rice per unit root under As exposure, was different between rice genotypes, and found in the order of As tolerant Triguna (134) > IR-36 (71.5) > PNR-519 (53) > sensitive IET-4786 (29). However, the grain As concentration (μg g^−1^dw) order was IR-36 (1.5) > Triguna (1) > PNR-519 (0.5) > IET-4786 (0.3). They concluded that most of the essential amino acids (EAAs) metabolites such as valine, metheionine, leucine, alanine, and nonessential amino acids (NEAAs) *viz.* histidine, alanine, proline, glutamic acid, and cysteine increased in most of the rice genotypes during As(V) exposure. Further to validate this finding a field experiment was conducted, determining the amino acid profile of sixteen rice genotypes differing in grain As accumulation, grown at three sites with different soil As concentrations in West Bengal and India. Grain As accumulation negatively correlated with EAAs which were more prominent in high As accumulating rice genotypes (HAARGs). Conversely, NEAAs showed an increase in low As accumulating rice genotypes (LAARGs) but a decrease in HAARGs. EAAs like isoleucine, leucine, valine, phenylalanine, and tyrosine also decreased in most of the genotypes (Dwivedi et al., 2012). Some other amino acids for example proline, glutamic acid, aspartic acid, and alanine also increased during As(V) stress in *Spinacia oleracea* (Pavlík et al., 2010). Among stress responsive amino acids, proline is a much studied molecules and can function as an osmolyte, free radical scavenger and also protects the cell membrane against damage. The level of proline has also been observed to be elevated in *O. sativa* during As (III) stress (Mishra and Dubey, 2006). The S-containing amino acid, cysteine, plays a central role in As detoxification, as it is a primary metabolite for synthesis of GSH and PCs. The cysteine content increased in some aquatic plants such as *H. verticillata*, rootless plant *C. demersum*, and crop plants *B. juncea*, *O. sativa* during As stress (Srivastava et al., 2007, 2009; Mishra et al., 2008; Tripathi et al., 2012).

### Other metabolites

Some low molecular antioxidant like ascorbate (AsA) and dehydroascorbate (DAsA), which work as non-enzymatic antioxidants in the glutathione-ascorbate cycle for free radical scavenging, were also analyzed in some plants during As(V) exposure. As(V) exposure caused an increase in the ratio of AsA/DAsA in *P. vitatta, P. ensiformis, H. verticillata, and O. sativa* (Singh et al., 2006; Srivastava et al., 2011; Tripathi et al., 2012) indicating the significant role of ascorbate for As induced stress amelioration. Malondialdehyde (MDA), the byproduct of lipid peroxidation was also increased during As(V) expsoure in *P. vittata*, *P. ensiformis*, *H. verticillata*, *C. demersum*. Nitric oxide, a signaling molecule was also found to be induced during As(V) stress condition in *H. verticillata*, *O. sativa*, and *A. thaliana* (Srivastava et al., 2011; Tripathi et al., 2012; Leterrier et al., 2012). The protection provided by polyamines against oxidative stress has been proposed to involve scavenging free radicals (Drolet et al., 1986) and the reduction of lipid peroxidation (Borrell et al., 1997). Mascher et al. (2002) demonstrated that levels of polyamines viz., spermidine, spermine increased only at lower doses but diamine increased at higher doses during As(V) stress in red clover (*Trifolium pretense*). Another study concluded that redox state and energetic equilibrium analyzed in terms of ATP/ADP NADH/NAD, NADPH/NADP, GSH/GSSG, and AsA/DAsA ratios, were found to be altered due to As toxicity in *H. verticillata* (Srivastava et al., 2011). Hence, variation in metabolite profiling during As exposure in different plant species signify that plants modulate their metabolome to respond against As stress.

## Future prospects

**Figure 1:**
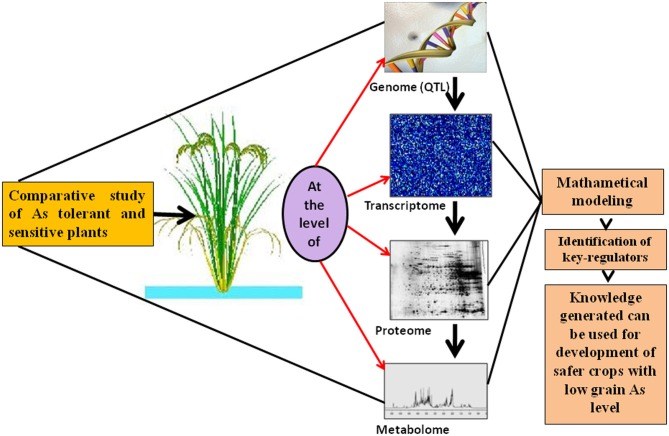
**Omics of As accumulation and tolerance: comparative study of As tolerant and sensitive plants at various levels such as genome, transcriptome, proteome, and metabolome to generate information to develop low grain As crops using breeding and molecular tools**.

As presents a health hazard to human populations world-wide due to its mobilization and accumulation in plant parts. As accumulation and homeostasis require the co-ordination of several processes working simultaneously to regulate uptake, long-distance transport, and distribution of metalloid to different cells and tissues. In the last few years, various QTLs as well as genes including those encoding transporters, genes mediating As accumulation, vacuolar sequestration, and distribution breakthroughs in As speciation with a diverse range of advanced techniques opening a new and unheralded insight to cellular speciation, such as micro-XAS and coupled HPLC-ICP-MS - ESI-MS. The evaluation of transcriptomic, proteomic, and metabolomic analyses indicate that thiol peptides like glutathione and PCs play a central role in As detoxification, as well as various antioxidant defense system response against As induced oxidative stress. However, not many studies have been carried out to study global change in term of transcriptome, proteome, and metabolome. A comparative evaluation of proteome, transcriptome, and metabolomic approaches in tolerant and sensitive varieties of plants such as rice and other plants, including *Arabdopsis*, may offer huge opportunities for the deeper understanding to develop As tolerant plants, including safer crops for human consumption (Figure 1). Further, studies pertaining to transcriptional responses show expression of several genes with unclear or unknown biological functions, providing future targets for plant Arsenomics research. However, the QTLs analysis in various As stressed plant species provide an insight into the genetic basis of As uptake and accumulation and will be useful for molecular breeding for As tolerance in rice. Epistatic interaction for grain As appear promising to reduce the health risk due to this carcinogen. Understanding of these omics approaches which will lead to Arsenomics and use of information generated could help to breed plants with low As in edible plant parts, along with the species of As present being of low toxicity. Therefore, future research should focus on filling gaps in our knowledge, taking advantages of modern analytical tools and a combination of different omics approaches for enhanced As phytoremediation and development of As tolerant crops with safer grain As levels.

### Conflict of interest statement

The authors declare that the research was conducted in the absence of any commercial or financial relationships that could be construed as a potential conflict of interest.

## Abbreviations

As: arsenic
As (V): arsenate
As(III): arsenite
AR: arsenate reductase
AsA: ascorbate
DAsA: dehydroascorbate
APX: ascorbate peroxidase
CAT: catalase
CS: cysteine synthase
DHAR: dehydroascorbate reductase
FBAII: fructose bisphosphate aldolase II
FBPase: fructose 1,6 bisphosphatase
GR: glutathione reductase
GST: glutathione-S-transeferase
GPX: guaiacol peroxidase
GSH: reduced glutathione
JA: jasmonic acid
MDHAR: monodehydroascorbate reductase
NIPs-: nodulin26-like intrinsic proteins
PC: phytochelatin
PCS: phytochelatin synthase
POD: peroxidase
Prx: peroxiredoxin
PGK: phosphoglycerate kinase
ROS: reactive oxygen species
SAM: S-adenosyl-L-methionine
SAMS: S-adenosylmethionine synthetase
SOD: superoxide dismutase
Trx: thioredoxin
TK: transketolase
γ-EC: γ-glutamyl cysteine
γ-ECS: γ-glutamyl cysteine synthatase.
